# Persistent Inequalities in Child Undernutrition in Cambodia from 2000 until Today

**DOI:** 10.3390/nu8050297

**Published:** 2016-05-16

**Authors:** Valérie Greffeuille, Prak Sophonneary, Arnaud Laillou, Ludovic Gauthier, Rathmony Hong, Rathavuth Hong, Etienne Poirot, Marjoleine Dijkhuizen, Frank Wieringa, Jacques Berger

**Affiliations:** 1 JRU NUTRIPASS IRD-SupAgro-UM, 911 av Agropolis, Montpellier 34000, France; gauthier.ludo@hotmail.fr (L.G.); franck.wieringa@ird.fr (F.W.); jacques.berger@ird.fr (J.B.); 2 National Nutrition Program, Maternal and Child Health Center, No. 31A, Rue de France (St. 47), Phnom Penh 12202, Cambodia; sophonprak@gmail.com; 3 United Nations Children’s Emergency Fund, Maternal, Newborn and Child Health and Nutrition Section, No. 11 Street 75, Phnom Penh 12202, Cambodia; alaillou@unicef.org (A.L.); rhong@unicef.org (R.H.); epoirot@unicef.org (E.P.); 4 ICF International, 530 Gaither Road, Suite 500, Rockville, MD 20850, USA; rathavuth.hong@icfi.com; 5 Department of Human Nutrition, Copenhagen University, Rolighedsvej 26, Frederiksberg 1958, Denmark; madijkhuizen@gmail.com

**Keywords:** stunting, wasting, anemia, children, inequity, Cambodia, Southeast Asia

## Abstract

The study assessed the trends of nutritional status of children under age five in Cambodia over four DHS surveys from 2000 to 2014 and the contribution of socioeconomic and demographic factors to its changes. Undernutrition was a public health problem in all surveys. Despite consistent improvement over the years, stunting still affected 32.5% of children in 2014. Wasting prevalence did not improve since 2005 and affected 9.6% of children under five in 2014. Low wealth and mother education; and rural residence contributed to undernutrition. In 2014; wealth status was the main socioeconomic factor associated with undernutrition and the nutritional status of children was strongly related to that of their mothers. Anemia prevalence was high and after a decrease between 2000 and 2005 remained at 45%. The prevalence of overweight was less than 10% and did not change over the years despite an increasing trend in the richest households of urban areas. Persistent inequalities in child undernutrition call for action, giving priority to the most vulnerable households to ensure availability and access to nutrient-rich foods for women and children through nutrition-sensitive and nutrition-specific programs. The recent increase of overweight in the richest populations must also be considered in Cambodian health policies.

## 1. Introduction

Undernutrition is a global health problem associated with increased morbidity and mortality in children [1,2]. Long-term undernutrition results in impaired health and physical and cognitive development, and lower productivity of populations. In the context of economic and demographic transition, several developing countries also encounter nutritional transitions with the increasing prevalence of overweight and obesity. This aspect of malnutrition has consequences on health through its association with non-communicable diseases [3]. Therefore, developing countries where problems of under- and overnutrition coexist suffer a so-called double burden of malnutrition.

Several efforts have been made to reduce malnutrition, resulting in a global decrease of undernutrition over the past decades [4]. Nevertheless, despite these efforts, improving nutritional status remains a challenge. Several actions were identified as efficient in preventing malnutrition. These actions concern both direct and indirect determinants, e.g., access to health services or increased food security as well as basic causes of malnutrition like poverty. Several interventions were implemented locally with proven beneficial effects on nutritional status. However, these actions need to be scaled up to reach the populations that need them most. Indeed, the magnitude of progress in the coverage of intervention influences the extent to which inequity between wealth groups can be reduced; the more coverage increases, the more inequity has a chance to decrease [5]. The prevalence of stunting and wasting among children under five has declined globally but still remains prevalent in South Asia and Africa [4]; these global improvements may hide disparities between child nutritional outcomes and improvements unevenly affecting populations according to their living area and socioeconomic characteristics. The poorest people living in rural areas are often the most at risk for undernutrition [4,6,7]. Gender inequality is also reported, even if its effects are less severe than economic inequality, with boys often being more affected by stunting and mortality before five years old than girls [4]. The picture of global trends and inequalities is different for overweight and obesity. Black *et al*. [4] reported a 54% increase in the prevalence of overweight in children under five years old from 1990 to 2011. Socioeconomic inequalities are less pronounced than for undernutrition, and overweight tends to affect the richest population groups more than the poorest.

Although a lot of developing countries succeeded in reducing undernutrition, most of them failed to significantly reduce the inequalities, *i.e.*, the differences in the prevalence of nutrition problems between subgroups of the population differing by their socioeconomic or demographic characteristics [8]. Yet the analysis of inequalities trends could help to define more efficient policies to reduce inequalities and to reach the most vulnerable subgroups of population.

In Cambodia, the great economic growth over the past two decades was not beneficial in the same manner to every subgroup of the population [9]. For example, the analysis of Kinnon *et al*. [9] showed that in Cambodia in 2010 the risk of neonatal death was 85% lower for infants belonging to the wealthier class compared to the lower class. These authors also reported that, of the 24 countries studied, Cambodia is one of the rare countries where wealth and educational inequalities in neonatal mortality rates increased.

The aims of the present study were to analyze the trends in nutritional status (anthropometry and anemia) of children under five years in Cambodia and to assess the effect of inequality (age, gender, mother’s education, living area, and wealth index) on nutrition in this population. To focus on the most recent situation of the country we also modeled the contribution of different socioeconomic factors and living areas in the prevalence of childhood undernutrition in 2014.

## 2. Material and Methods

### 2.1. Data Sources

This study used data from the Cambodia Demographic Health surveys (CDHS) conducted in 2000, 2005, 2010, and 2014 (Macro international Inc., Opinion Research Corporation (ORC Macro), Caverlton, MD, USA). The DHS surveys collect information on household demographic and socioeconomic characteristics and on child anthropometry, child feeding practices, and child health in a nationally representative sample [10,11,12,13]. The surveys were based on stratified samples selected at two stages, and each reporting domain was separated into rural and urban areas.

### 2.2. Indicators Used

The household wealth index was constructed by principal component analysis, as described by Filmer and Pritchett [14]. The wealth index is a composite measure of a household’s living standard that was calculated using data consistently available over time. It gathers information regarding accessibility and type of water, sanitation facilities, materials used for housing construction, type of fuel used for cooking, and ownership of selected assets such as a radio, television, refrigerator, *etc*.

Anthropometric measurements were collected from children under five years in a subsample of households. Children’s heights were measured to the nearest 1 mm. The nutritional status of children was defined by the height-for-age, weight-for-height, and weight-for-age *z*-scores calculated according to the Child Growth Standard of the World Health Organization (WHO) using the igrowup macro designed for STATA [15]. *z*-scores below −2 for length/height-for-age, weight-for-length/height, and weight-for-age were defined as stunting, wasting, and underweight, respectively. Overweight was defined as BMI-for-age *z*-score > 2. To insure the accuracy of the data, extreme values were excluded from the analysis: weight-for-age *z*-score < −6 or >5; length/height-for-age *z*-score < −6 or >6; weight-for-length/height *z*-score < −5 or >5; BMI-for-age *z*-score < −5 or >5. These excluded values represented 7.9%, 7.0%, 6.2%, and 5.6%, respectively, of the total study sample in 2000, 2005, 2010, and 2014. Anemia was defined as a hemoglobin concentration below 110 g/L.

Diarrhea was reported for every child with diarrhea in the two weeks preceding the survey. The children who suffered from Acute Respiratory Infection (ARI) in the two weeks preceding the survey were also examined. ARI is defined as a cough and rapid breathing.

### 2.3 Statistical Analysis

Analysis was performed using STATA v11 (Stata Corp LP, College Station, TX, USA), taking into account the complex sampling design of DHS surveys using the STATA’s svyset function. Standard errors were estimated using the Taylor series linearization method, which incorporates sampling weights and uses variance formulas appropriate to the DHS sample design. *Z*-tests on weighted percentages made it possible to compare the results of the different surveys. Associations between malnutrition indicators (stunting, wasting, underweight, overweight, and anemia) and socioeconomic factors of inequality consistently available over the surveys (age, gender, mother’s education, living area, and wealth index) were assessed using logistic regressions. For each survey, the relative inequality between subgroups was assessed by calculating the Odd Ratio between extreme categories indicated in parentheses in the tables. Trends in malnutrition indicators were also assessed for each sub-group of the population, by calculating the absolute difference in prevalence observed for each survey period, and logistic regressions were run to determine if these were statistically significant. We defined differences as statistically significant when *p* < 0.05. We reported prevalence with standard errors, Odds Ratios (OR) with 95% confidence intervals, and differences in prevalence over years.

To model the nutritional status of children in 2014 as a function of their socioeconomic characteristics, we used multivariate logistic regression. Variables in the model were selected through a backward stepwise conditional approach. Variables not significant in the model (*p >* 0.05) were excluded. The covariates used to build the model were: age in months (0–6 months, 6–11 months, 12–17 months, 18–24 months, 24–35 months, 36–47 months, 48–59 months) gender, maternal education (none, primary, secondary), living area (urban/rural), wealth index (poorest, poorer, middle, richer, richest), maternal BMI (low (<18.5 kg/m^2^), normal (≤18.5 kg/m^2^ and <25 kg/m^2^), overweight (≥25 kg/m^2^)), occurrence of diarrhea or acute respiratory infection in the two weeks preceding the survey (except for stunting analysis), the time since the preceding birth from the same mother (months), which corresponds to the age difference with the nearest sibling, and mother’s tobacco use. Age of children and gender remained in the model even if non-significant. The analysis includes all children under five years old surveyed in Cambodia DHS 2014 except for anemia, which only concerns children six months or older. Collinearity between variables was checked by calculating the Variance Inflation Factor (VIF) for each explanatory variable, as described before [16]. The VIF was calculated for each model and values were all <2.5 (comprised between 1.00 and 1.48), indicating no problem of collinearity. Both *p*-values and OR (95% CI) were reported in the table.

## 3. Results

Table 1 presents the characteristics of children from the four surveys. The male/female ratio was, as expected, close to 50/50 in each survey. The rural/urban ratio was approximately 6/1 in 2000 and decreased to 2.8/1 in the 2014 survey. The percentage of mothers without education decreased from 3/10 to approximately 1/10 over time. The mean age of children was not significantly different over the four studies. In contrast, mean height and weight of all children and in males and females increased progressively and significantly over time. Consequently, height-for-age and weight-for-age indices improved significantly from 2000 to 2014, while weight-for-height *z*-scores and BMI-for-age *z*-scores did not change significantly over the four surveys despite an improvement between 2000 and 2005.

Concerning the nutritional indicators, stunting represented a public health problem in all surveys: very high in boys from 2000 to 2010 and high in 2014 according to the WHO classification [17]; very high in girls in 2000 and high from 2005 to 2014 (Table 2). Stunting was similarly prevalent in both sexes over time, except it was significantly higher in males in 2005. Stunting prevalence decreased significantly over the study period for both sexes. In each survey, the risk of being stunted was significantly higher in children whose mothers had no education than for those of mothers with secondary education or higher (the prevalence of stunting was intermediate in women with primary education). The prevalence of stunting was also significantly higher in children living in rural areas in all four surveys than in those living in urban areas. From 2005 to 2014 the stunting prevalence was about twice as high in children in the poorest wealth quintile compared to children in the richest quintile, with the prevalence of stunting decreasing from the poorest to the richest households.

Wasting was a public health problem over time: very high in both sexes in 2000, medium in both sexes in 2005, high in both sexes in 2010, and again medium in both sexes in 2014 (Table 3), according to the WHO classification. Wasting was similarly prevalent in both sexes over time and not significantly different according to the education of mothers. Until the 2014 survey, wasting did not differ significantly between urban and rural area, whereas in 2014 rural prevalence was significantly higher than urban ones. The risk of being wasted was significantly higher in children in the poorest wealth quintile compared to children in the richest quintile in 2005 and 2014.

The prevalence of underweight (Table 4) was not significantly different between the sexes over time, representing a very high public health problem in 2000 and a high public health problem thereafter. The prevalence of underweight decreased significantly over the study period. The risk of being underweight was significantly higher in children whose mother had no education than in those with mothers with secondary education (the prevalence of underweight was intermediate in women with primary education). The risk of being underweight was also significantly higher in children living in rural areas compared to urban areas in the 2000, 2010, and 2014 surveys. The prevalence of underweight was about twice as high in the poorest quintile compared to the richest quintile, with the prevalence of underweight decreasing form the poorest to the richest households.

The prevalence of overweight (OW) did not change significantly over the study period (it was always below 10%) and was significantly lower in females than in males in 2005 and 2014 (Table 5). OW increased over time in urban areas and decreased in rural areas; the prevalence of OW in urban areas was almost twice that of rural areas in 2010 and 2014. In 2014, belonging to the richest category was a risk factor for OW compared to the poorest.

Anemia prevalence was a significant severe public health problem in all four surveys and the risk of being anemic was significantly higher in males compared to females in 2005 and 2010 (Table 6). The prevalence of anemia decreased significantly over the years in both males and females, especially between 2000 and 2010, and remained stable thereafter. In the 2000 and 2005 surveys, the prevalence of anemia was significantly higher in children whose mothers had no education (compared to secondary education) and no more statistically different thereafter. In contrast, the differences between rural and urban areas were significant only in 2010 and 2014, with the children living in rural areas being more at risk than those in urban areas. The differences between the poorest and the richest quintiles were significant whatever the survey. The poorest were the most at risk, with the prevalence of anemia decreasing from the poorest to the richest households.

**Figure 1:**
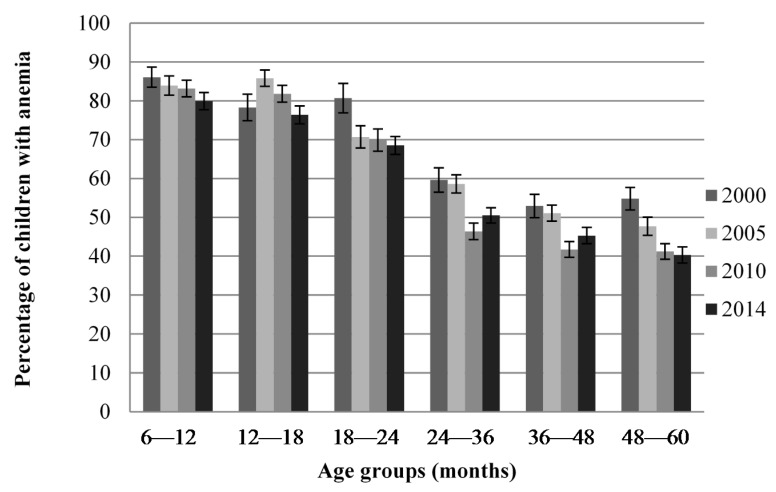
Percentage of children with anemia by age groups for each survey (% ± standard error).

Figure 1 shows that anemia prevalence was higher in 6–18-month-old children compared to older children in all four surveys but was still a public health problem in all age groups and in all surveys. Data were also available for 0–6-month-old children in the 2000 survey and was 60.6% compared to 86.1% in 6–12-month-old children (data not shown).

The multivariate analysis indicated that, in the 2014 survey, the significant factors contributing to undernutrition, *i.e.*, stunting, wasting, and underweight, were birth weight, BMI of mothers, and wealth index, with the risk of being stunted, wasted, and underweight higher in children who had a low birth weight, a mother with low BMI, and the lowest category of wealth (Table 7). Age was a contributing factor for stunting and underweight, with a higher risk in older children. Being younger, living in urban settings, and a higher BMI of mother were risk factors for overweight in children. Being younger, living in a rural area, having a mother with low BMI, and belonging to the poorest wealth quintile were associated with anemia. Wasting and stunting were risk factors for having anemia.

## 4. Discussion

Undernutrition was a public health problem in Cambodian children under five years of age in all four surveys conducted from 2000 to 2014. Stunting, underweight, and anemia were the most worrisome nutritional problems in both male and female children, affecting one third, one fourth, and more than half of children, respectively [17]. The prevalence of these three nutritional problems decreased consistently over the 14-year period. Wasting also decreased from 2000 to 2010 but slightly increased again thereafter to affect one child in 10, thus still representing a mild health problem in 2014. Globally, undernutrition was higher in the poorest children, children living in rural areas and, except for wasting, in children with mothers with no education in all four surveys. Overweight prevalence was less than 10% and inequalities towards overweight between wealth quintiles and living areas appeared only recently, in 2010 and 2014, respectively.

Most of the low- and middle-income countries in Southeast Asia (SEA) suffer from stunting and its prevalence in Cambodia in the 2010 and 2014 surveys (39% and 33%, respectively) were in the range of other SEA countries—from 23.2% in Vietnam [18] to 35% in Myanmar [19] and 44% in Laos (44%) [20]. In all surveys from 2000 to 2014, stunting prevalence was significantly higher in children living in rural areas and having a mother with no education, and had an inverse relationship with wealth quintiles.

The higher prevalence of stunting in the poorest wealth categories is in line with a recent analysis of the inequalities in child undernutrition in 80 countries [8]. This underlines the inequality of access to economic development for Cambodian households, which probably results in inequality of access to an adequately nutritious diet for children during the critical window of the first two years of age. Indeed, access to a diversity of nutrient-rich foods is a key link in the relationship between higher income and lower prevalence of stunting [21]. In addition, it is worth noting that, in the four surveys, the prevalence of stunting among the poor was approximately and consistently twice as high as among the richest. These findings indicate that the significant global decrease in stunting prevalence since 2000 was not accompanied by a narrowing of the wealth gap. However, in the 2014 survey stunting still affected about one fifth of the richest.

No decrease in inequality for living area toward stunting was observed since 2000 because stunting decreased in a similar trend in both rural and urban areas. Similar changes were observed in other SEA countries, where the height of children and stunting improved in both rural and urban areas from 1985 to 2011 [6] but the poorest people living in rural areas remain the most at risk for undernutrition [7,21]. In the most recent Cambodian survey in 2014, the prevalence of stunting was 11% higher in rural areas but a multivariate model indicated that the living area was not significantly associated with stunting, suggesting that the differences in stunting prevalence between rural and urban areas were related to the difference in household wealth status in these two areas instead.

The oldest children were more at risk of being stunted, probably because growth retardation is a cumulative process that develops mainly during the two first years of age [22]. Stunting prevalence was consistently lower in children with mothers having the highest level of education. This result is consistent with findings from cross-sectional studies carried out in different contexts [23] and in Asia [24]. In Cambodia, the inequalities toward stunting related to the mother’s education decreased significantly between 2005 and 2014, mainly due to a significant higher decrease of stunting prevalence in the category with no education, whereas no significant changes were observed in the category of higher education after 2005. These different trends of stunting according to mother’s education might explain why, in 2014, the multivariate model indicated that mothers’ education was not associated with stunting.

The prevalence of wasting decreased by half between 2000 and 2005 but remained a medium-high health problem thereafter, affecting approximately 10% of children in 2014, placing Cambodia in the scope of several SEA countries—where wasting prevalence ranges from 4.1% in Vietnam to 12.1% in Indonesia [25]. In Cambodia, wasting was strongly related to the socioeconomic status of the household in 2005 and in 2014, with the poorest households exhibiting the highest prevalence. From 2000 to 2014 wasting prevalence was neither significantly different between boys and girls, nor between mothers with different education levels. Inequality between residence areas appeared only in 2014 and became significantly higher in rural areas. Multivariate analysis showed that, in 2014, the wealth index is the only socioeconomic factor significantly linked to wasting prevalence. The other contributing factors were linked to the nutritional status of mothers instead (low BMI of mothers and low birth weight). This high prevalence of wasting in Cambodia is of concern because wasting has a direct and immediate impact on the mortality risk in children and was recently shown to be a stronger predictor of mortality than stunting or underweight [26]. The origin of wasting, often described as “acute malnutrition”, is reported to be a sudden and drastic lack of nutrients due to sickness and/or lack of food availability (including the hunger gap season, drought, flood, and displaced populations) [27]. However, most of the risk factors for wasting are also associated with stunting, and wasting can also became a “chronic” problem deeply linked to stunting when these situations are frequent or accumulate [28]. Thus these two forms of malnutrition share common causal factors, suggesting that some interventions could address both problems.

In Cambodia, the prevalence of underweight was approximately 10% less in each survey than the prevalence of stunting. This can be explained by the fact that some stunted children have a weight-for-height higher than the reference for their age. For instance, in the most recent survey in 2014, 7.7% of stunted children had weight-for-height *z*-scores higher than 1 (3.5% higher than 2) so that 92% of these children were not underweight. A recent analysis noted that the positive and significant correlations between underweight and stunting observed in all regions of the world prove their close relationship even if these two indicators describe different physiological and biological processes [26]. The authors indicate that even in regions with a high level of wasting, similar to the prevalence found in the four surveys in Cambodia, the correlation of underweight with stunting was consistent; and that in Asian sub-regions such as SEA, underweight accounted for more than 70% of stunting. It was thus not surprising that, in the four surveys, underweight mainly followed the same pattern of inequality as stunting, with the prevalence being significantly higher in the poorest children, those living in rural areas, and those with low education mothers.

The regression analysis carried out in the 2014 survey provided information on the causes of wasting, stunting, and underweight, showing similarities in their determinants. In addition to wealth status, low BMI of mothers was a contributing factor to undernutrition of children as well as low birth weight. Similar findings for wealth status and low BMI as explanatory factors for wasting and stunting have been showed in two studies in India [27,29], suggesting that intergenerational associations in wasting and stunting are not only driven by maternal intrauterine influences but also by undernutrition in the household. A previous study suggested that the reduction of child stunting in Cambodia was mainly linked to improvements in household wealth, sanitation, parental education, birth spacing, and reduction of maternal tobacco use [30]. It has been demonstrated that direct interventions such as improving complementary foods and feeding practices of infants, micronutrient supplementation and fortification, and reduction of disease burden can decrease the prevalence of undernutrition [31,32] but that improvement of underlying determinants, such as poverty, poor education, disease burden, and lack of women’s empowerment also have to be improved to eliminate malnutrition in the long term [33]. Regarding results for Cambodia, improving wealth status of households and of mothers, as well as nutritional status and empowerment of women, would be the keys to preventing stunting and wasting by diminishing the prevalence of small-for-gestational-age infants. Furthermore, dietary diversity and consumption of animal products were shown to be protective factors against stunting in Cambodia [34]. Thus investing in agricultural programs to increase the availability and accessibility of nutrient-rich food in Cambodia for all population groups but especially for adolescent girls and women before and during pregnancy would have a positive impact on child nutritional status.

The prevalence of overweight was of concern in all surveys, and after a decrease between 2000 and 2005 remained stable around 7% thereafter. These findings are in line with data from the SEA region. In Vietnam, two recent surveys indicated a prevalence of about 7% in children under five [18,35], while a study in Indonesia reported a prevalence of 6.2% in urban settings and 3.2% in rural settings for children aged six months to two years [36]. In Thailand in 2011, 4.2% of children aged six months to three years in urban areas and 7.1% in rural areas were overweight or obese [37], and a study in Malaysia indicated 8% of overweight or obesity in children 0–13 years old [38].

However, it is worth noting that, in Cambodia, the prevalence of overweight did not increase over the last 10 years, contrary to what is observed in many countries over the world, even in developing countries [4,39]. Recently, Black *et al*. [4] reported a 54% increase in global overweight prevalence in children from 1990 to 2011 and considered that this trend of increase is expected to continue in most parts of the word, even if their projections for 2025 suggest the plausibility of either an increase or a decrease in overweight prevalence in Asia.

It is interesting to note that whereas the global prevalence of overweight did not increase over the last 15 years, inequalities for overweight between socioeconomic subgroups appeared in 2014, with a higher prevalence in the richest wealth group compared to other wealth groups. Moreover, the prevalence of OW in 2014 was higher in boys than in girls. We also observed a widening of inequalities between rural and urban areas, with a constant increase of overweight in urban areas from 2005. Thus, in 2014, the urban prevalence of overweight was double that in 2005. Furthermore, in the 2014 survey, overweight was significantly related to low age, mother with higher BMI, and residence in urban settings. This link between maternal high BMI and children’s overweight was already reported by several authors and maternal obesity is identified as one of the strongest risk factors for child obesity, since the child’s eating habits resemble those of the family diet patterns [40,41]. The level of education of the mother was not related to child overweight whereas overweight in women was related to their low education level [42].

These results suggest that effective strategies to prevent and control overweight and obesity in children should take into account the fact that prevalence increases more quickly in urban areas and in the richest populations and that programs improving household feeding practices in the most-at-risk populations should benefit both mothers and their offspring.

Anemia represented a severe public health problem in Cambodia without any significant improvement since 2000; in 2014 55% of children were still anemic. Anemia affected the youngest children more, from six months to two years old, during the critical period of immunity development and mutation from breastmilk feeding to family-like feeding. This strong link between age and anemia prevalence was confirmed in the multivariate model of 2014, which indicated that anemia most affected the poorest people and those living in rural areas. The gap between rural and urban areas widened after 2005, while anemia inequality for wealth did not change significantly. In contrast, the inequalities that existed in the different mother education subgroups disappeared from 2010. The failure to significantly decrease anemia prevalence over the years called into question the main possible causes of anemia in Cambodia. Micronutrient deficiencies and especially iron deficiency, as well as vitamin A and vitamin B12 deficiencies, are one of the most commonly encountered causes of anemia in the world. The Cambodian DHS reported that in 2014 only 3%, 11%, and 11% of children had iron deficiency, and vitamin A and B12 deficiencies, respectively. However, the prevalence of these micronutrient deficiencies followed the same picture as that for anemia, with children between six and 24 months of age being affected more than the older ones. Furthermore, anemia was associated with stunting and wasting. These findings suggest that anemia partly reflected the general undernutrition of children, especially in the first two years of age, and probably also the poor quality and quantity of diet including complementary feeding practices. Nevertheless, these results also suggest that other causes such as the high prevalence of hemoglobinopathies in this country, which have been indicated in several studies [43,44], might also contribute to anemia.

## 5. Conclusions

Undernutrition, especially stunting and anemia, and to a lesser extent wasting, were still worrying public health problems in Cambodian children under five in 2014. The significant decrease of stunting since 2010 shows that Cambodia, which is on the edge of becoming a middle-income country, has made significant efforts to improve the nutrition and health of its population. However, these efforts have to be maintained and priority must be directed to the most vulnerable households and to integrating nutrition-sensitive interventions to complement direct nutrition interventions. Moreover, interventions must tackle malnutrition of both children and women of reproductive age before and during pregnancy. Anemia prevalence has not changed significantly in 15 years and non-nutritional causes are likely to be the main contributors to the high prevalence of anemia in Cambodia. A better understanding of the etiology of anemia in Cambodia is needed to set up appropriate intervention strategies. Overweight did not increase globally in children under five in the last 15 years, but there was a trend towards an increase in overweight in the richest families living in urban areas that must serve as a warning to Cambodian stakeholders and policy makers.

## Figures and Tables

**Table 1:** Characteristics of children included in the analysis from the Cambodian DHS surveys of 2000, 2005, 2010, and 2014.

		2000			2005			2010			2014			Year Effect
		*n*	Mean/Prevalence	S E	*n*	Mean/Prevalence	S E	*n*	Mean/Prevalence	S E	*n*	Mean/Prevalence	S E	*p* Value
Age (months)	Male	1807	**30.5**	0.5	1941	**29.8**	0.4	2082	**30.5**	0.4	2504	**29.3**	0.4	0.249
	Female	1742	**30.6**	0.5	1978	**29.6**	0.4	2015	**29.9**	0.4	2431	**29.8**	0.4	0.962
Height (cm)	Male	1809	**82.8**	0.4	1941	**82.9**	0.3	2085	**84.0**	0.3	2507	**83.9**	0.3	<0.001
	Female	1744	**81.5**	0.4	1978	**81.8**	0.3	2016	**82.1**	0.3	2435	**82.8**	0.3	<0.001
Weight (kg)	Male	1809	**10.53**	0.09	1941	**10.81**	0.07	2085	**10.96**	0.07	2507	**11.00**	0.08	<0.001
	Female	1744	**10.07**	0.08	1978	**10.23**	0.08	2016	**10.28**	0.08	2435	**10.43**	0.08	0.010
Height-for-age *z*-score	Male	1809	**−1.87**	0.05	1941	**−1.86**	0.04	2085	**−1.65**	0.04	2507	**−1.40**	0.04	<0.001
	Female	1744	**−1.87**	0.05	1978	**−1.66**	0.04	2016	**−1.65**	0.04	2435	**−1.42**	0.04	<0.001
Weight-for-age *z*-score	Male	1807	**−1.69**	0.04	1941	**−1.46**	0.03	2082	**−1.42**	0.03	2504	**−1.23**	0.03	<0.001
	Female	1742	**−1.65**	0.04	1978	**−1.43**	0.03	2015	**−1.44**	0.03	2431	**−1.30**	0.03	0.001
Weight-for-height *z*-score	Male	1806	**−0.90**	0.04	1939	**−0.61**	0.03	2082	**−0.71**	0.03	2504	**−0.64**	0.03	0.172
	Female	1741	**−0.76**	0.04	1977	**−0.65**	0.03	2014	**−0.69**	0.03	2430	**−0.67**	0.03	0.597
BMI-for-age *z*-score	Male	1807	**−0.68**	0.04	1941	**−0.38**	0.03	2082	**−0.51**	0.03	2504	**−0.48**	0.03	0.234
	Female	1742	**−0.62**	0.04	1978	**−0.52**	0.03	2015	**−0.56**	0.03	2431	**−0.56**	0.03	0.338
hemoglobin concentration (g/L)	Male	794	**102**	1	1683	**104**	0	1922	**106**	0	2278	**106**	0	0.027
	Female	786	**105**	1	1668	**106**	0	1829	**108**	0	2190	**108**	0	0.104
Gender (%)	Male	1882	**50.7**	0.8	1941	**49.5**	0.8	2085	**50.8**	0.8	2508	**50.7**	0.7	0.492
	Female	1829	**49.3**	0.8	1978	**50.5**	0.8	2016	**49.2**	0.8	2435	**49.3**	0.7	
Residence (%)	Urban	531	**14.3**	0.6	784	**20.0**	0.6	1063	**25.9**	0.7	1322	**26.7**	0.6	0.933
	Rural	3180	**85.7**	0.6	3135	**80.0**	0.6	3038	**74.1**	0.7	3621	**73.3**	0.6	
Orphan status (%)	Non Orphan	3604	**97.1**	0.3	3819	**97.4**	0.3	4021	**98.0**	0.2	4845	**98.0**	0.2	0.100
	Orphan	107	**2.9**	0.3	100	**2.6**	0.3	80	**2.0**	0.2	98	**2.0**	0.2	
Mother’s education (%)	None	640	**35.6**	1.1	1114	**29.8**	0.8	847	**22.2**	0.7	618	**13.9**	0.5	<0.001
	Primary	938	**52.2**	1.2	2077	**55.5**	0.8	1985	**52.0**	0.8	2241	**50.4**	0.8	0.404
	Secondary+	220	**12.2**	0.8	550	**14.7**	0.6	988	**25.9**	0.7	1586	**35.7**	0.7	<0.001
Wealth quintile (%)	Poorest	1015	**27.4**	0.7	1158	**29.5**	0.7	1060	**25.8**	0.7	1288	**26.1**	0.6	0.962
	Poorer	844	**22.7**	0.7	892	**22.8**	0.7	789	**19.2**	0.6	896	**18.1**	0.6	0.046
	Middle	765	**20.6**	0.7	678	**17.3**	0.6	678	**16.5**	0.6	828	**16.8**	0.5	0.468
	Richer	627	**16.9**	0.6	651	**16.6**	0.6	714	**17.4**	0.6	817	**16.5**	0.5	0.252
	Richest	460	**12.4**	0.5	540	**13.8**	0.6	860	**21.1**	0.6	1113	**22.5**	0.6	<0.001

**Table 2:** Prevalence of stunting in children in the four surveys according to their social characteristics.

Characteristics	% (Sd. Err)				Trends over Time **		
	2000	2005	2010	2014	2000–2014	2005–2014	2010–2014
CHILD’S SEX							
Male	49.1 (1.5)	45.3 (1.5)	40.2 (1.4)	32.9 (1.3)	−16.2 *	−12.4 *	−7.3 *
Female	48.6 (1.0)	39.3 (1.3)	38.1 (1.5)	32.2 (1.3)	−16.4 *	−7.1 *	−5.9 *
OR (Male:Female) (95% CI)	1.02 (0.88–1.18)	1.27 * (1.09–1.49)	1.09 (0.95–1.26)	1.03 (0.88–1.20)			
MOTHER’S EDUCATION							
None	56.8 (2.5)	51.7 (2.1)	46.4 (2.2)	37.8 (2.8)	−19.0 *	−13.9 *	−8.6 *
Primary	47.5 (1.9)	42.7 (1.4)	40.1 (1.5)	34.0 (1.3)	−13.5 *	−8.7 *	−6.1 *
Secondary+	37.0 (3.8)	26.9 (2.3)	30.1 (1.9)	27.1 (1.6)	−9.9 *	0.2	−3
OR (Secondary:None) (95% CI)	0.44 * (0.31–0.65)	0.34 * (0.26–0.46)	0.50 * (0.39–0.64)	0.61 * (0.46–0.80)			
RESIDENCE							
Urban	41.1 (2.6)	34.8 (2.8)	28.4 (2.0)	23.4 (1.5)	−17.7 *	−11.4 *	−5.0 *
Rural	50.1 (1.2)	43.3 (1.2)	41.1 (1.3)	34.0 (1.1)	−16.1 *	−9.3 *	−7.1 *
OR (Urban:Rural) (95% CI)	0.69 * (0.55–0.87)	0.70 * (0.57–0.90)	0.57 * (0.46–0.71)	0.59 * (0.49–0.72)			
WEALTH QUINTILE							
Poorest	56.8 (2.0)	52.5 (2.0)	50.2 (2.0)	40.9 (2.0)	**−15.9**	**−11.6**	**−9.3**
Poorer	50.9 (2.1)	47.5 (2.2)	43.2 (2.0)	36.0 (1.9)	**−14.9**	**−11.5**	**−7.2**
Middle	50.4 (2.5)	44.3 (2.3)	39.3 (2.6)	32.4 (2.1)	**−18**	**−11.9**	**−6.9**
Richer	45.8 (2.7)	33.2 (2.4)	32.1 (2.5)	29.1 (2.3)	**−16.7**	−4.1	−3
Richest	31.7 (2.4)	25.7 (2.4)	25.0 (1.9)	20.5 (1.7)	**−11.2**	−5.2	−4.5
OR (Richest:Poorest) (95% CI)	0.35 * (0.27–0.46)	0.31 * (0.23–0.42)	0.33 * (0.26–0.42)	0.37 * (0.29–0.48)			
Total	48.8 (1.1)	42.2 (1.1)	39.2 (1.1)	32.5 (1.0)	−16.3 *	−9.7 *	−6.7 *

* Significant differences (*p* < 0.05); ** Trends over time indicate absolute differences between survey years in each subgroup of the population.

**Table 3:** Prevalence of wasting in children in the four surveys according to their social characteristics.

Characteristic	% (Sd. Err)				Trends over Time **		
	2000	2005	2010	2014	2000–2014	2005–2014	2010–2014
CHILD’S SEX							
Male	17.8 (1.2)	8.6 (0.8)	11.2 (0.8)	9.8 (0.8)	−8.0 *	1.2	−1.4
Female	15.1 (1.1)	8.3 (0.8)	10.2 (0.8)	9.4 (0.7)	−5.7 *	1.1	−0.8
OR (Male:Female) (95% CI)	1.21 (0.98–1.50)	1.03 (0.78–1.37)	1.12 (0.90–1.39)	1.04 (0.82–1.32)			
MOTHER’S EDUCATION							
None	17.3 (2.2)	9.8 (1.2)	11.1 (1.2)	12.3 (2.0)	−5.0	2.5	1.2
Primary	16.2 (2.5)	8.4 (0.8)	11.5 (0.9)	9.4 (0.8)	−6.8 *	1.0	−2.1
Secondary+	17.9 (3.5)	7.7 (1.3)	9.4 (1.3)	9.5 (1.0)	−8.4 *	1.8	0.1
OR (Second.:None) (95% CI)	1.04 (0.61–1.78)	0.76 (0.50–1.18)	0.83 (0.57–1.22)	0.75 (0.48–1.18)			
RESIDENCE							
Urban	15.0 (2.0)	9.3 (1.2)	11.5 (1.5)	7.7 (0.9)	−7.3 *	−1.6	−3.8 *
Rural	16.7 (0.9)	8.3 (0.6)	10.6 (0.7)	9.9 (0.6)	−6.8 *	1.6	−0.7
OR (Urban:Rural) (95% CI)	0.87 (0.62–1.24)	1.13 (0.82–1.56)	1.10 (0.80–1.52)	0.76 * (0.58–0.99)			
WEALTH QUINTILE							
Poorest	17.1 (1.5)	11.5 (1.3)	11.8 (1.2)	11.5 (1.1)	−5.6 *	0.0	−0.3
Poorer	15.5 (1.6)	9.2 (1.2)	9.1 (1.1)	11.3 (1.3)	−4.2 *	2.1	2.2
Middle	15.5 (1.5)	6.7 (1.2)	11.8 (1.6)	8.5 (1.2)	−7.0 *	1.8	−3.3
Richer	16.6 (1.9)	6.5 (1.1)	10.9 (1.4)	8.6 (1.2)	−8.0 *	2.1	−2.3
Richest	18.2 (2.4)	6.5 (1.3)	9.6 (1.4)	7.2 (1.0)	−11 *	0.7	−2.4
OR (Richest:Poorest) (95% CI)	1.08 (0.73–1.59)	0.53 * (0.33–0.85)	0.79 (0.53–1.17)	0.60 * (0.42–0.86)			
Total	16.5 (0.9)	8.4 (0.6)	10.7 (0.6)	9.6 (0.6)	−6.88	1.22	−1.08

* Significant differences (*p* < 0.05); ** Trends over time indicates absolute differences between years of survey in each subgroup of population.

**Table 4:** Prevalence of underweight in children in the four surveys according to their social characteristics.

Characteristic	% (Sd. Err)				Trends over Time **		
	2000	2005	2010	2014	2000–2014	2005–2014	2010–2014
CHILD’S SEX							
Male	38.8 (1.45)	29.6 (1.3)	27.3 (1.3)	23.3 (1.1)	−15.5 *	−6.3 *	−4.0 *
Female	38.5 (1.1)	26.9 (1.3)	28.9 (1.4)	25.1 (1.2)	−13.4 *	−1.8	−3.8 *
OR (Male:Female) (95% CI)	1.01 (0.86–1.20)	1.14 (0.98–1.33)	0.92 (0.77–1.10)	0.91 (0.76–1.07)			
MOTHER’S EDUCATION							
None	43.3 (2.8)	32.6 (1.9)	33.7 (2.2)	29.3 (2.8)	−14.0 *	−3.3	−4.4
Primary	37.4 (2.0)	29.3 (1.3)	28.7 (1.4)	25.1 (1.2)	−12.3 *	−4.2 *	−3.6
Secondary+	30.3 (3.3)	19.5 (2.1)	20.2 (1.8)	21.4 (1.4)	−8.9 *	1.9	1.2
OR (Secondary:None) (95% CI)	0.57 * (0.39–0.83)	0.50 * (0.37–0.68)	0.50 * (0.37–0.67)	0.66 * (0.48–0.89)			
RESIDENCE							
Urban	31.1 (2.7)	28.5 (2.7)	17.5 (1.6)	16.1 (1.3)	−15.0 *	−12.4 *	−1.4
Rural	39.9 (1.1)	28.1 (1.1)	29.9 (1.1)	25.5 (1.0)	−14.4 *	−2.6	−4.4 *
OR (Urban:Rural) (95% CI)	0.68 * (0.52–0.88)	1.01 (0.77–1.34)	0.50 * (0.39–0.63)	0.56 * (0.45–0.69)			
WEALTH QUINTILE							
Poorest	43.7 (1.9)	34.8 (1.9)	35.9 (2.0)	30.9 (1.9)	−12.8 *	−3.9	−5
Poorer	41.4 (2.1)	32.4 (2.0)	31.7 (1.9)	26.9 (1.6)	−14.5 *	−5.5 *	−4.8 *
Middle	37.2 (2.4)	27.4 (2.1)	27.5 (2.2)	23.3 (1.7)	−13.9 *	−4.1	−4.2
Richer	36.1 (2.3)	24.7 (2.1)	23.9 (2.1)	22.3 (2.0)	−13.8 *	−2.4	−1.6
Richest	30.1 (2.6)	16.4 (1.9)	16.6 (1.8)	14.7 (1.4)	−15.4 *	−1.7	−1.9
OR (Richest:Poorest) (95% CI)	0.55 * (0.41–0.74)	0.37 * (0.27–0.50)	0.35 * (0.26–0.48)	0.39 * (0.29–0.51)			
Total	38.6 (1.03)	28.2 (0.98)	28.0 (0.98)	24.2 (0.88)	−14.4 *	−4.0 *	−3.8 *

* Significant differences (*p* < 0.05); ** Trends over time indicate absolute differences between survey years in each subgroup of the population.

**Table 5:** Prevalence of overweight in children in the four surveys according to their social characteristics.

Characteristic	% (Sd. Err)				Trends over Time		
	2000	2005	2010	2014	2000–2014	2005–2015	2010–2014
CHILD’S SEX							
Male	9.3 (0.8)	9.4 (0.9)	7.5 (0.7)	8.3 (0.7)	−1	−1.1	0.8
Female	9.0 (0.8)	6.2 (0.7)	7.2 (0.7)	6.5 (0.6)	−2.5 *	0.3	−0.7
OR (Male:Female) (95% CI)	1.02 (0.79–1.34)	1.58 * (1.19–2.11)	1.04 (0.80–1.36)	1.30 * (1.02–1.67)			
MOTHER’S EDUCATION							
None	7.6 (1.2)	8.4 (1.1)	8.5 (1.3)	6.2 (1.1)	−1.4	−2.2	−2.3
Primary	8.4 (1.1)	7.6 (0.8)	6.7 (0.7)	6.8 (0.7)	−1.6	−0.8	0.1
Secondary+	11.2 (2.6)	6.9 (1.4)	7.8 (1.1)	8.4 (0.9)	−2.8	1.5	0.6
OR (Second.:None) (95% CI)	1.54 (0.82–2.88)	0.80 (0.48–1.34)	0.90 (0.57–1.42)	1.38 (0.90–2.13)			
RESIDENCE							
Urban	9.0 (1.8)	6.5 (1.5)	10.4 (1.2)	11.9 (1.3)	2.9	5.4 *	1.5
Rural	9.2 (0.7)	7.9 (0.7)	6.8 (0.5)	6.7 (0.5)	−2.5 *	−1.2	−0.1
OR (Urban:Rural) (95% CI)	0.97 (0.61–1.54)	0.80 (0.47–1.36)	1.59 * (1.18–2.15)	1.89 * (1.41–2.52)			
WEALTH QUINTILE							
Poorest	8.1 (1.0)	8.1 (1.3)	9.0 (1.1)	6.9 (0.9)	−1.2	−1.2	−2.1
Poorer	8.4 (1.2)	8.1 (1.2)	7.0 (1.2)	6.7 (0.9)	−1.7	−1.4	−0.3
Middle	8.8 (1.3)	5.2 (1.0)	4.8 (0.9)	6.6 (1.0)	−2.2	1.4	1.8
Richer	11.0 (1.5)	8.6 (1.4)	6.8 (1.3)	5.7 (1.0)	−5.3 *	−2.9	−1.1
Richest	10.5 (2.1)	8.6 (1.7)	8.7 (1.2)	11.4 (1.5)	0.9	2.8	2.7
OR (Richest:Poorest) (95% CI)	1.33 (0.79–2.24)	1.08 (0.63–1.82)	0.96 (0.65–1.41)	1.73 * (1.17–2.57)			
Total	9.2 (0.61)	7.7 (0.60)	7.3 (0.50)	7.4 (0.50)	−1.8	−0.3	0.1

* Significant differences (*p* < 0.05); ** Trends over time indicate absolute differences between survey years in each subgroup of the population.

**Table 6:** Prevalence of anemia in children in the four surveys according to their social characteristics.

Characteristic	% (Sd. Err)				Trends over Time **		
	2000	2005	2010	2014	2000–2014	2005–2014	2010–2014
CHILD’S SEX							
Male	65.7 (2.0)	64.2 (1.7)	57.4 (1.4)	56.7 (1.4)	−9.0 *	−7.5 *	−0.7
Female	61.1 (1.9)	59.5 (1.4)	52.5 (1.5)	54.2 (1.4)	−6.9 *	−5.3 *	1.7
OR (Male:Female) (95% CI)	1.15 (0.91–1.46)	1.22 * (1.02–1.46)	1.20 * (1.02–1.40)	1.11 (0.96–1.28)			
MOTHER’S EDUCATION							
None	70.1 (2.5)	68.5 (2.1)	57.3 (2.1)	56.6 (2.7)	−13.5 *	−11.9 *	−0.7
Primary	62.5 (2.0)	62.3 (1.4)	56.9 (1.6)	58.8 (1.5)	−3.7	−3.5	1.9
Secondary+	52.1 (4.0)	52.5 (3.2)	51.8 (2.1)	52.4 (1.8)	0.3	−0.1	0.6
OR (Secondary:None) (95% CI)	0.46 * (0.31–0.70)	0.51 * (0.37–0.69)	0.80 (0.63–1.01)	0.84 (0.66–1.08)			
RESIDENCE							
Urban	57.3 (3.8)	59.7 (4.1)	44.7 (2.1)	43.4 (1.9)	−13.9 *	−16.3 *	−1.3
Rural	64.4 (1.5)	62.2 (1.1)	56.9 (1.2)	57.4 (1.2)	**−7.0**	−4.8 *	0.5
OR (Urban:Rural) (95% CI)	0.74 (0.53–1.03)	0.90 (0.64–1.27)	0.61 * (0.50–0.75)	0.57 * (0.48–0.68)			
WEALTH QUINTILE							
Poorest	66.7 (2.7)	68.0(2.0)	60.1 (2.0)	64.9 (1.8)	−1.8	−3.1	4.8
Poorer	67.1 (3.0)	68.0 (2.2)	58.2 (2.2)	57.9 (2.2)	−9.2 *	−10.1 *	−0.3
Middle	61.3 (3.2)	57.5 (2.5)	56.6 (2.2)	55.6 (2.2)	−5.7	−1.9	−1
Richer	62 (3.9)	55.8 (2.7)	53.2 (2.5)	49.0 (2.5)	−13.0 *	−6.8	−4.2
Richest	48.7 (4.7)	52.8 (3.2)	44.3 (2.4)	44.6 (2.1)	−4.1	−8.2 *	0.3
OR (Richest:Poorest) (95% CI)	0.47 * (0.30–0.74)	0.52 * (0.38–0.72)	0.53 * (0.41–0.67)	0.43 * (0.34–0.55)			
Total	63.5 (1.39)	61.8 (1.11)	55.0 (1.10)	55.5 (1.10)	−8.0	−6.3	0.5

* Significant differences (*p* < 0.05); ** Trends over time indicate absolute differences between survey years of in each subgroup of the population.

**Table 7:** *p*-values and regression coefficients of contributing factors to stunting, wasting, overweight, and anemia in the 2014 DHS survey.

	Stunting *n* = 3886		Wasting *n* = 3886		Underweight *n* = 3886		Overweight *n* = 4302		Anemia *n* = 3798	
	*p*-Value	OR ** (95% CI)	*p*-Value	OR ** (95% CI)	*p*-Value	OR ** (95% CI)	*p*-Value	OR ** (95% CI)	*p*-Value	OR ** (95% CI)
Age in months *	<0.001	1.22 (1.16–1.28)	Ns	-	<0.001	1.23 (1.16–1.29)	<0.001	0.83 (0.78–0.89)	<0.001	0.66 (0.63–0.70)
Gender (ref = male) *	Ns.	-	Ns.	-	Ns.	-	Ns.	-	Ns.	-
Wealth index	<0.001	0.83 (0.78–0.89)	0.015	0.89 (0.82–0.98)	<0.001	0.84 (0.7–0.90)	-	-	<0.001	0.85 (0.79–0.91)
Living area (ref = rural)	-	-	-	-	-	-	<0.001	2.15 (1.56 2.96)	0.034	0.77 (0.60 0.98)
BMI of mother	<0.001	0.63 (0.52–0.78)	<0.001	0.56 (0.41–0.76)	<0.001	0.49 (0.40–0.60)	0.044	1.33 (1.01–1.76)	0.017	0.81 (0.6–0.96)
Low birth weight	<0.001	2.12 (1.51–2.98)	<0.001	2.30 (1.49–3.55)	<0.001	2.59 (1.83–3.66)	-	-	-	-
Wasting									0.039	1.35 (1.02–1.78)
Stunting									<0.001	1.46 (1.22–1.75)

* Age and gender were included in the model even if non-significant; ** OR indicated the nature of the effect of explanatory variables on the dependent variable when it changes from one category to the next.
