# Correction: Greffeuille, V.; et al. Persistent Inequalities in Child Undernutrition in Cambodia from 2000 Until Today. *Nutrients* 2016, *8*, 297

**DOI:** 10.3390/nu8100459

**Published:** 2016-09-30

**Authors:** Valérie Greffeuille, Prak Sophonneary, Arnaud Laillou, Ludovic Gauthier, Rathmony Hong, Rathavuth Hong, Etienne Poirot, Marjoleine Dijkhuizen, Frank Wieringa, Jacques Berger

**Affiliations:** 1 JRU NUTRIPASS IRD-SupAgro-UM, 911 av Agropolis, Montpellier 34000, France; gauthier.ludo@hotmail.fr (L.G.); franck.wieringa@ird.fr (F.W.); jacques.berger@ird.fr (J.B.); 2 National Nutrition Program, Maternal and Child Health Center, No. 31A, Rue de France (St. 47), Phnom Penh 12202, Cambodia; sophonprak@gmail.com; 3 United Nations Children’s Emergency Fund, Maternal, Newborn and Child Health and Nutrition Section, No. 11 street 75, Phnom Penh 12202, Cambodia; alaillou@unicef.org (A.L.); rhong@unicef.org (R.H.); epoirot@unicef.org (E.P.); 4 ICF International, 530 Gaither Road, Suite 500, Rockville, MD 20850, USA; rathavuth.hong@icfi.com; 5 Department of Human Nutrition, Copenhagen University, Rolighedsvej 26, Frederiksberg 1958, Denmark; madijkhuizen@gmail.com

We would like to submit the following as a correction to our recently published paper [1] because of the use of the wrong cut-off for overweight. The prevalence of overweight has been corrected all over Table 5, and subsequently the column concerning overweight in Table 7.

These corrections induced a few and limited changes in the text of the results and discussion sections. As a consequence of this correction, the following sentences should be corrected:

## Results

On page 4

The prevalence of overweight (OW) did not change significantly from 2005 to 2014 being equal or lower than 2.2% (Table 5). Significant differences were observed according to child’s sex and wealth index in 2014 only, prevalence being higher in males than in females and lower in the poorest than in richest households. OW increased over time in urban areas and decreased in rural areas with a higher prevalence of OW in urban areas from 2010.

On page 5

The multivariate analysis indicated that, in the 2014 survey, the significant factors contributing to undernutrition, i.e., stunting, wasting, and underweight, were birth weight, BMI of mothers, and wealth index, with the risk of being stunted, wasted, and underweight higher in children who had a low birth weight, a mother with low BMI, and the lowest category of wealth (Table 7). Age was a contributing factor for stunting and underweight, with a higher risk in older children. Being younger, **being a male and belonging to the richest quintile** were risk factors for overweight in children. Being younger, living in a rural area, having a mother with low BMI, and belonging to the poorest wealth quintile were associated with anemia. Wasting and stunting were risk factors for having anemia.

## Discussion

On page 13

Undernutrition was a public health problem in Cambodian children under five years of age in all four surveys conducted from 2000 to 2014. Stunting, underweight, and anemia were the most worrisome nutritional problems in both male and female children, affecting one third, one fourth, and more than half of children, respectively. The prevalence of these three nutritional problems decreased consistently over the 14-year period. Wasting also decreased from 2000 to 2010 but slightly increased again thereafter to affect one child in 10, thus still representing a mild health problem in 2014. Globally, undernutrition was higher in the poorest children, children living in rural areas and, except for wasting, in children with mothers with no education in all four surveys. Overweight prevalence **did not exceed 2.2% over the last ten years** and inequalities towards overweight between **gender and** wealth quintiles appeared only recently **in 2014.**

On page 14

The prevalence of overweight **stayed stable and didn’t exceed 2.2%** in all surveys **from** 2005. **This prevalence of overweight is lower than the prevalence in other countries of the** SEA region. In Vietnam, two recent surveys indicated a prevalence of about 7% in children under five, while a study in Indonesia reported a prevalence of 6.2% in urban settings and 3.2% in rural settings for children aged six months to two years. In Thailand in 2011, 4.2% of children aged six months to three years in urban areas and 7.1% in rural areas were overweight or obese, and a study in Malaysia indicated 8% of overweight or obesity in children 0–13 years old.

On page 14

It is interesting to note that whereas the global prevalence of overweight did not increase over the last 15 years, inequalities for overweight between socioeconomic subgroups appeared in 2014, with a higher prevalence in the richest wealth group compared to other wealth groups. Moreover, the prevalence of OW in 2014 was higher in boys than in girls. We also observed **significant** inequalities between rural and urban areas **from 2010, due to a constant** increase of overweight in urban areas from 2005. Thus, in 2014, the urban prevalence of overweight was **more than** double that in 2005. Furthermore, in the 2014 survey, overweight was significantly related to low age, **being a male and belonging to the richest wealth quintile. No significant link was identified between** maternal high BMI and children’s overweight **contrasting with several studies that reported** maternal obesity as one of the strongest risk factors for child obesity. The level of education of the mother was not related to child overweight whereas overweight in women was related to their low education level.

These changes have no material impact on the conclusions of our paper. We apologize for any inconvenience caused to our readers.

## Figures and Tables

**Table 5:** Prevalence of overweight in children in the four surveys according to their social characteristics.

	% (Sd. Err)				Trends over Time **		
	2000	2005	2010	2014	2014–2000	2014–2005	2014–2010
CHILD’S SEX							
Male	3.0 (0.5)	2.2 (0.4)	2.2 (0.4)	2.7 (0.4)	−0.3	0.5	0.5
Female	3.6 (0.6)	1.5 (0.3)	1.7 (0.3)	1.6 (0.4)	−2.0 *	0.1	−0.1
OR (Male:Female)	0.84 (0.53–1.33)	1.48 (0.85–2.56)	1.3 (0.73–2.3)	1.77 * (1.04–2.99)			
MOTHER EDUCATION							
None	3.0 (0.1)	2.0 (0.5)	1.4 (0.5)	1.9 (0.6)	−1.1	−0.1	0.5
Primary	2.8 (0.1)	1.8 (0.3)	2.0 (0.3)	2.4 (0.4)	−0.2	0.6	0.4
Secondary+	1.4 (0.1)	1.4 (0.5)	2.6 (0.7)	2.2 (0.5)	0.8	0.8	−0.4
OR (Second:None)	0.46 (0.11–1.97)	0.69 (0.27–1.77)	1.94 (0.76–4.93)	1.25 (0.57–2.74)			
RESIDENCE							
Urban	2.5 (0.7)	1.4 (0.7)	3.1 (0.8)	3.2 (0.8)	0.7	1.8	0.1
Rural	3.4 (0.4)	1.9 (0.3)	1.7 (0.3)	2.0 (0.3)	−1.4 *	0.1	0.3
OR (Urban:Rural)	0.73 (0.38–1.4)	0.76 (0.27–2.17)	1.85 * (1.01–3.38)	1.6 (0.89–2.87)			
WEALTH QUINTILE							
Poorest	2.9 (0.8)	2.4 (0.6)	1.9 (0.5)	1.2 (0.3)	−1.7 *	−1.2 *	−0.7
Poorer	2.8 (0.7)	2.2 (0.6)	1.8 (0.6)	1.9 (0.5)	−0.9	−0.3	0.1
Middle	2.7 (0.7)	0.6 (0.3)	1.1 (0.4)	2.8 (0.7)	0.1	2.2 *	1.7 *
Richer	4.2 (1.0)	1.4 (0.6)	1.9 (0.6)	1.7 (0.5)	−2.5 *	0.3	−0.2
Richest	4.5 (1.2)	2.2 (0.8)	3.2 (0.9)	3.6 (0.9)	−0.9	1.4	0.4
OR (Richest:Poorest)	1.56 (0.71–3.42)	0.9 (0.37–2.17)	1.71 (0.76–3.82)	3.11 * (1.48–6.52)			
Total	3.4 (0.4)	2.2 (0.3)	2.0 (0.3)	2.2 (0.3)	−1.2 *	0.0	0.2

* Significant differences (*p* < 0.05); ** Trends over time indicate absolute differences between survey years in each subgroup of the population.

**Table 7:** *p*-values and odds ratio of contributing factors to stunting, wasting, overweight, and anemia in the 2014 DHS survey.

	Stunting *n* = 3886		Wasting *n* = 3886		Underweight *n* = 3886		Overweight *n* = 4302		Anemia *n* = 3798	
	*p*-Value	OR ** (95% CI)	*p*-Value	OR ** (95% CI)	*p*-Value	OR ** (95% CI)	*p*-Value	OR ** (95% CI)	*p*-Value	OR ** (95% CI)
Age in months *	<0.001	1.22 (1.16–1.28)	Ns.	-	<0.001	1.23 (1.16–1.29)	<0.001	0.77 (0.69–0.86)	<0.001	0.66 (0.63–0.70)
Gender (ref = female) *	Ns.	-	Ns.	-	Ns.	-	0.031	1.76 (1.05–2.94)	Ns.	-
Wealth index	<0.001	0.83 (0.78–0.89)	0.015	0.89 (0.82–0.98)	<0.001	0.84 (0.7–0.90)	0.038	1.22 (1.01–1.47)	<0.001	0.85 (0.79–0.91)
Living area (ref = rural)	-	-	-	-	-	-	-	-	0.034	0.77 (0.60–0.98)
BMI of mother	<0.001	0.63 (0.52–0.78)	<0.001	0.56 (0.41–0.76)	<0.001	0.49 (0.40–0.60)	-	-	0.017	0.81 (0.6–0.96)
Low birth weight (ref = normal birth weight)	<0.001	2.12 (1.51–2.98)	<0.001	2.30 (1.49–3.55)	<0.001	2.59 (1.83–3.66)	-	-	-	-
Wasting									0.039	1.35 (1.02–1.78)
Stunting									<0.001	1.46 (1.22–1.75)

* Age and gender were included in the model even if non-significant (Ns.); ** OR indicated the nature of the effect of explanatory variables on the dependent variable when it changes from one category to the next.
